# Visual Teaching System Design of University Political Video Course Based on Network Node Algorithm

**DOI:** 10.1155/2022/9849888

**Published:** 2022-09-09

**Authors:** Dan Zheng, Rong Huang, Qi Chen

**Affiliations:** Department of Information Engineering, Qinhuangdao Vocational and Technical College, Qinhuangdao 066100, Hebei, China

## Abstract

With the continuous development of wireless sensor networks in the fields of industrial measurement and control, medical monitoring, and intelligent buildings, the demand for network performance is also increasing. Conventional MAC protocols used in wireless sensor networks can no longer meet the low power consumption, interference suppression, high reliability, and high throughput communication requirements of these applications. With the changes in related visual media in education, from wood boards and cards in the age of visual media to slides and projections in the age of audio-visual media, as well as the extensive development of various visual tools, visual teaching systems are all in terms of technology, environment, mode, and meaning. Visual education research in the development of video courses first needs to understand the educational function of the smart classroom, including the related concepts, research progress, and key functions of the smart classroom. At the same time, it is also necessary to clarify the theoretical principles and expression methods of video course development and their effectiveness in professional training. Finally, we can combine the advantages of the two educational institutions to design applied research and apply it to visual education in smart classrooms. This is exactly where the educational philosophy of the university political curriculum of visualized higher education policy plays an important role in running the university political curriculum of higher education policy. In the context of multiculturalism, how to deal with the new problems and new situations of the current ideals and concepts of college students, how to grasp the main problems, and how to solve the problems are a topic worthy of continuous discussion and summary.

## 1. Introduction

Broadcast communication is an important form of communication in wireless sensor networks [1]. Many applications in wireless sensor networks are based on broadcast communication, such as routing query and time synchronization, executing data query, updating network node programs, and so on [2]. In a single-channel environment, all nodes in the network are included on the same channel. The broadcast information sent from the node can be received by the node in the radio communication area using the broadcast characteristics of the radio wave [3]. Therefore, broadcast communication can be realized quickly and efficiently. However, in a multichannel environment, the nodes in the network are distributed on different channels, and radio wave transmission characteristics (WBA) cannot be realized. If a node sends broadcast information on a specific channel, nodes on other channels cannot receive the information. On the other hand, wireless sensor networks usually introduce node sleep states to save energy [4]. Different nodes have different wake-up times. Therefore, when a node sends a broadcast packet, the sleeping node cannot receive it. Since the nodes in the wireless network are distributed on different channels and wake up at different times, it is difficult to achieve broadcast communication in the multichannel environment of the wireless sensor network [5]. In the visual teaching system, visual technology provides a processing method from abstract to concrete, provides educational activities with intuitive functions, and supports breakthroughs in educational forms and methods [6]. When explaining visual education, some people also mentioned the role of technology in the process of visualizing the teaching system. They think that visual education is the use of simulation and simulation in the process of perception, cognition, imagination, and reasoning through the use of computer software and multimedia materials [7]. Visualization is shown in the classroom as much as possible. This article combines the characteristics of video course development and training with the design of the visual environment of the future classroom and proposes the characteristics of the visual education activities in the future classroom environment [8]. Based on the guidance of activity theory and the basis of existing research, the design elements and design process of university politics and video course development activities in the future classroom environment are proposed. Design elements include education content analysis, acceptance analysis, education goal analysis, activity tasks, activity organization forms, levels of support for visualization, rules, activity links, comments, etc. Each element is analyzed and explained in detail, and a framework for visual education activities in university political courses in the future teaching environment will be designed.

## 2. Related Work

The literature introduces the future classroom environment and system structure where the activity elements are introduced into the visual education activities. We propose the design process and design elements of the visual education activities in the future classroom environment, and carefully analyze the combination of learning framework theory and cognitive structure theory [9]. The different elements of activity design put forward a framework for designing visual education activities in the classroom environment in the future [10]. The literature introduces the application and structural fields of wireless sensor networks and summarizes the impact of the requirements of communication functions on wireless sensor networks according to the characteristics of wireless sensor networks [11]. Introduced multichannel communication technology and analysis of the problems that must be solved to realize multichannel communication [12]. The literature describes the advantages and disadvantages of multichannel protocols. This document proposes a protocol that is a solution to the problems of serious node conflicts, node loss, and unfair communication conflicts in existing protocols. Based on the existing protocol, in order to reduce the degree of node conflicts, a time-slicing mechanism is introduced [13]. The random time slice method avoids the generation of lost nodes and guarantees the fairness of communication between nodes. And through the simulation platform, the designed protocol and the original protocol were compared and tested [14]. The literature introduces channel interference in the actual application of wireless sensor networks, the requirements for wireless sensor networks for channel detection, and the requirements for wireless sensor network nodes for spectrum collection. Based on the analysis of spectrum sensing technology in the field of wireless cognition, a collaborative spectrum sensing method based on finite-dimensional random matrix theory is proposed [15]. The detection threshold is checked according to the DCN distribution function formula of the random matrix contained in the scheme, and the DCN distribution function expression of the random matrix of any dimension is derived. The literature introduces the security issues of the existing time synchronization protocol [16]. According to the characteristics of the bad attack time synchronization protocol, the fault-tolerant network time synchronization protocol-FTTSP protocol for wireless sensor networks has been developed. The protocol uses a detection mechanism to prevent incorrect synchronization information sent by malicious nodes. Based on information fusion, it makes full use of the synchronization information of each neighboring node and uses a random weighted average algorithm to eliminate slow attacks.

## 3. Wireless Sensor Network and Visual Teaching System Model Design

### 3.1. Energy Consumption Model of the Wireless Sensor Network

N sensor nodes are randomly distributed in the monitoring area, the length is *L* and the width is W. The location of the base station close to the monitoring area remains unchanged. Assume that the network status is as follows:

1. Homogeneous sensor nodes have unique IDs and will not move once they are distributed
2. The sensor node generates data packets at a speed of kbit/s and transmits them to the cluster head of the cluster
3. The cluster head directly transmits the data packet to the cluster head through one hop
4. The transmission radius of the node is adjustable
5. Calculate the approximate transmission distance based on the received signal strength between the two communication nodes

The node density *ρ* in the network can be expressed as

(1) $$\begin{array}{c} \rho =\frac{N}{L\times W}\text{.} \end{array}$$

According to the energy consumption formula, the energy consumption of any cluster can be analyzed. For cluster members, energy consumption is only represented by transmission energy consumption, which is determined by the size of the data packet and the distance between the node and the cluster head, as shown in the following formula:

(2) $$\begin{array}{c} E_{CM}=k\left({\pi \rho R}^{2}-1\right)\cdot \left(E_{elec\;}+\varepsilon_{fs\;}{\bar{d_{toCH}}}^{2}\right)\text{.} \end{array}$$

The expectation of the distance between the member node and the cluster head can be expressed as

(3) $$\begin{array}{c} E\left(d\right)=\bar{d_{\mathrm{toCH}}}=\frac{1}{{\pi R}^{2}}\cdot \iint \sqrt{\left(x^{2}+y^{2}\right)}dx\;dy\text{,}\\ =\frac{1}{{\pi R}^{2}}\cdot \iint r^{2}\mathrm{drd}\theta \text{,}\\ =\frac{1}{{\pi R}^{2}}\cdot \int_{0}^{2\pi }d\theta \int_{0}^{R}r^{2}\mathrm{dr}=\frac{2R}{3}. \end{array}$$

Further, formula (2) can be expressed as

(4) $$\begin{array}{c} E_{CM}=k\left({\pi \rho R}^{2}-1\right)\cdot \left(E_{elec\;}+\frac{4}{9}\varepsilon_{fs}R^{2}\right)\text{.} \end{array}$$

The energy consumption of the cluster head consists of three parts: receiving energy consumption, fusion energy consumption, and transmission energy consumption. Therefore, ECH can be expressed as

(5) $$\begin{array}{c} E_{CH}=k\left({\pi R}^{2}\rho -1\right)E_{elec\;}+{k\pi R}^{2}{\rho E}_{DA}+{kE}_{elec\;}+{k\varepsilon }_{f_{s}}d_{toBS\;}^{2}\text{,}\\ ={k\pi R}^{2}\rho \left(E_{elec\;}+E_{DA}\right)+{k\varepsilon }_{f_{s}}d_{toBS\;}^{2}\text{.} \end{array}$$

The energy consumption of a cluster can be expressed as

(6) $$\begin{array}{c} E_{cluster\;}=E_{CM}+E_{CH}\text{.} \end{array}$$

The minimum and maximum conflict radii of computing nodes are shown below. At the same time, the energy consumption of clusters in the network should be minimized to maximize the network cycle life.

(7) $$\begin{array}{c} \left\{\begin{array}{c} E_{cluster\;}^{{\mathrm{MAX}\;d}_{loBs\;}}\left(R_{\min}\right)=E_{cluster\;}^{\text{MIN }d{\;}_{osS\;}}\left(R_{\max}\right)\text{,}\\ \min\;\left(E_{cluster\;}^{{\mathrm{MAX}\;d}_{doBS}}\left(R\right)\right)\text{,} \end{array}\right.\\ E_{cluster\;}^{0}=\min\;\left(E_{cluster\;}^{\mathrm{MAX}\;d{\;}_{oBS\;}}\left(R\right)\right)\text{,}\\ =k\left({\pi \rho R}^{2}-1\right)\cdot \left(E_{elec\;}+\frac{4}{9}\varepsilon_{fs}R^{2}\right)\text{,}+{k\pi R}^{2}\rho \left(E_{elec\;}+E_{DA}\right)+{k\varepsilon }_{fs}d_{toBS}^{2}. \end{array}$$

The ideal cluster head is that when ordinary nodes join the cluster head, the communication power consumption is minimized, and the participation of ordinary nodes will not overload the cluster head nodes far away from the base station. The metrics for evaluating ideal cluster heads are shown below. An ordinary node stores all received broadcast packets and calculates the cost of each monitorable cluster head.

(8) $$\begin{array}{c} \cos\;t\left(i\right)=c\cdot \frac{d_{\mathrm{toCH}}^{i}}{\text{MAX}\left(d_{\mathrm{toCH}}\right)}+\left(1-c\right)\cdot \frac{d_{\mathrm{toBS}}^{i}-\mathrm{MNN}\left(d_{\mathrm{toBS}}\right)}{\mathrm{MAX}\left(d_{\mathrm{toBS}}\right)-\text{MIN}\left(d_{\mathrm{toBS}}\right)}\text{.} \end{array}$$

The LEACH algorithm determines whether each sensor node is the cluster head node of the current round. The decision-making method is based on the number of cluster heads on the network and how often nodes become cluster heads. The sensor node *i* generates a random number from 0 to 1 when determining the cluster head, its determination probability is PI (*t*). The threshold Pi (*t*) is defined as follows:

(9) $$\begin{array}{c} P_{i}\left(t\right)=\left\{\begin{array}{c} \frac{k}{N-k^{*}\left(r\;\mathrm{mod}\;\left(N/k\right)\right)};C_{i}\left(t\right)=1\text{,}\\ 0;C_{i}\left(t\right)=0\text{,} \end{array}\right. \end{array}$$

The EECS algorithm node determines the cluster head through competition. The node Pj calculates the cost of each audible cluster head node according to the following cost function:

(10) $$\begin{array}{c} \cos\;t\left(j,i\right)=\left(1-w\left(P_{j}\right)\right)\times f\left(P_{j},{\mathrm{CH}}_{i}\right)+w\left(P_{j}\right)\times g\left({\mathrm{CH}}_{i}\right)\text{.} \end{array}$$

Join the cluster head node with the smallest cost, where

(11) $$\begin{array}{c} f\left(P_{j},{\mathrm{CH}}_{i}\right)=\frac{d\left(P_{j},{\mathrm{CH}}_{i}\right)}{d_{f_{-}\max}}\text{,}\\ g\left({\mathrm{CH}}_{i}\right)=\frac{d\left({\mathrm{CH}}_{i},\mathrm{BS}\right)-d_{g_{-}\min}}{d_{g_{-}\max}-d_{g_{-}\min}}\text{,} \end{array}$$

where d_g_-max_ is the maximum monitorable distance between the node Pj and the cluster head node, and *d*_g_-max_ is the maximum monitorable distance between the node Pj between the cluster head node and the base station, the definition of w (Pj) can be calculated follows:

(12) $$\begin{array}{c} w\left(P_{j}\right)=c+\left(1-c\right)\sqrt{\frac{d\left(P_{j},\mathrm{BS}\right)}{d_{g_{-}\max}-d_{g_{-}\min}}}\text{.} \end{array}$$

*Life Cycle (NL).* There are many ways to define the life cycle, such as when the first node dies or half of the nodes in the network die.

ARE represents the average remaining energy of nodes in the network, which can be calculated as follows:

(13) $$\begin{array}{c} \text{ARE}=\frac{\;total\;residual\;energy\;of\;nodes\;}{\;mumber\;of\;nodes}\text{.} \end{array}$$

EBF is used to measure the energy balance of nodes, shown as follows:

(14) $$\begin{array}{c} \text{EBF}=\sqrt{\frac{1}{N}\overset{N}{\underset{i=0}{\sum }}{\left(E_{i}-E_{avg\;}\right)}^{2}}\text{.} \end{array}$$

AITC represents the average communication cost within the cluster.

**Figure 1:**
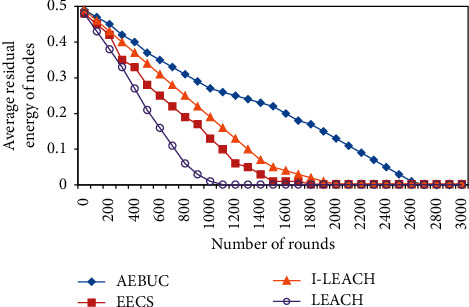
Average remaining energy of nodes.

Figure 1 shows that due to the uneven clustering strategy adopted by AEBCU, the cluster radius of cluster heads far away from the base station is reduced and the cluster radius of cluster heads close to the base station is increased, thereby offsetting the power consumption in the network and making AEBUC. The performance is significantly better than the other three algorithms.

**Figure 2:**
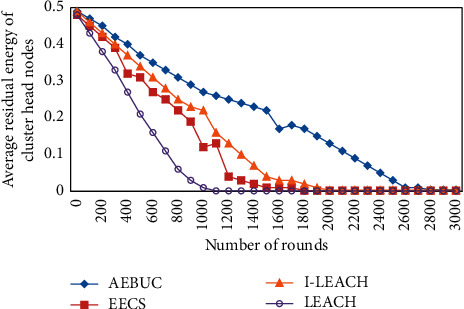
The average remaining energy of the cluster head node.

The average remaining energy of the cluster head node is shown in Figure 2.

### 3.2. Wireless Sensor Network Communication

**Figure 3:**
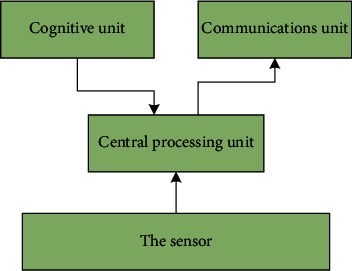
Multichannel communication block diagram of wireless cognitive sensor network.

In contrast to unlicensed frequency band congestion, many dedicated spectrum resources allocated to various existing wireless systems have varying degrees of time and space vacancy. Effective use of these idle resources will undoubtedly provide valuable communication resources for multichannel communication in wireless sensor networks, and this dynamic channel access corresponds to the event-driven communication characteristics of wireless sensor networks. In recent years, research based on cognitive radio has received more and more attention. The main idea of wireless cognitive sensor networks is to use different sensor technologies in wireless sensor networks to perceive what they are doing. You can adjust your own operating parameters to obtain the best-operating conditions. Multichannel communication uses cognitive radio spectrum detection technology to detect the state of the radio channel and adjust the communication strategy for multichannel communication to achieve the purpose of reliable communication with low power consumption.  Figure 3 shows the multichannel communication block diagram of the wireless cognitive sensor network.

**Figure 4:**
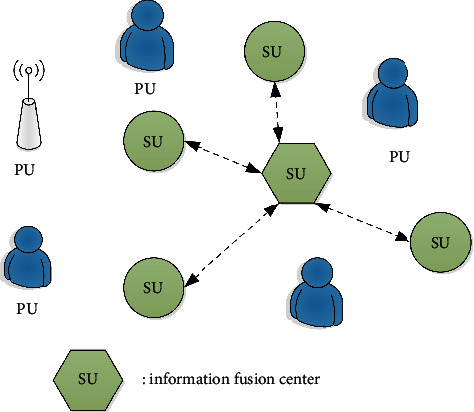
Cognitive radio model.

As shown in the cognitive radio model in Figure 4, the main user is an authorized user, who has the priority to use the radio channel. Secondary users can use channel resources for opportunistic access. For cognitive radio, secondary users must have spectrum acquisition capabilities. If the main user does not use the channel, the main user's channel resources will be used temporarily. When performing spectrum acquisition, secondary users can perform single-node acquisition based on the detected information, or send the detected information to the information fusion center to coordinate spectrum acquisition. Spectrum acquisition is an important technology in cognitive radio applications, many problems need to be solved in the following aspects: the signal-to-noise ratio of the primary user signal received from the secondary user is very low, but the secondary user determines whether there is a primary user based on the poor quality signal. The attenuation and time-varying characteristics of the radio channel signal will affect the main user signal and increase the difficulty of spectrum acquisition.

In the wireless cognitive sensor network, the wireless sensor node as the secondary user will not interfere with the normal operation of the primary user. Suppose that the node performs spectrum detection on the j-th signal on the channel:

(15) $$\begin{array}{c} r_{\mathrm{ij}}=\sqrt{\alpha }\cdot s_{\mathrm{ij}}+\sqrt{\beta }\cdot n_{\mathrm{ij}}\text{.} \end{array}$$

It is defined as

(16) $$\begin{array}{c} \mu =sup\left\{10\;\log_{10}\;\beta \right\}\text{.} \end{array}$$

Spectrum sensing is to perform the following hypothesis tests based on the received signal:

(17) $$\begin{array}{c} \begin{array}{c} \hbar_{0}:s_{\text{ij}}=0\text{,}\\ \hbar_{1}:s_{\text{ij}}\neq 0\text{,} \end{array} \end{array}$$

Assuming that the information fusion node used for spectrum collection collects information from K collection nodes, and each collection node has N collection data, then the information fusion node is a *K*×*N* random matrix H. With or without primary users, the random matrix H has different forms:

(18) $$\begin{array}{c} H=\overset{K}{\underset{i=1}{\sum }}\overset{N}{\underset{j=1}{\sum }}\left(r_{\mathrm{ij}}\cdot r_{\mathrm{ij}}^{H}\right). \end{array}$$

A random matrix can form a Wishart matrix W:

(19) $$\begin{array}{c} W={\mathrm{HH}}^{H},K\leq N\text{.} \end{array}$$

Obtain the eigenvalues of Wishart matrix W, arranged in descending order, the eigenvalues can be expressed as

(20) $$\begin{array}{c} \lambda_{1}\geq \lambda_{2}\geq \cdots \cdots \geq \lambda_{K}>0\text{.} \end{array}$$

The sum of all eigenvalues of the matrix is

(21) $$\begin{array}{c} T=\overset{K}{\underset{i=1}{\sum }}\lambda_{i}\text{.} \end{array}$$

Use the node to detect the data to form the Wishart matrix R:

(22) $$\begin{array}{c} R=\frac{1}{N}{\mathrm{HH}}^{H},K\leq N\text{,}\\ \gamma_{1}=\frac{{\left(\sqrt{N}+\sqrt{K}\right)}^{2}}{{\left(\sqrt{N}-\sqrt{K}\right)}^{2}}\cdot \left(1+\frac{{\left(\sqrt{N}+\sqrt{K}\right)}^{-2/3}}{{\left(\sqrt{\mathrm{NK}}\right)}^{1/6}}F_{1}^{-1}\left(1-P_{f}\right)\right)\text{.} \end{array}$$

### 3.3. Visual Teaching

In the context of the development of visual education, visual education has developed with the related changes in visual media education. Visual education has changed in terms of technology, environment, mode, and meaning. In particular, with the development of visualization technology and cognitive science, the current concept of visualization education is not limited to the visualization of symbols such as text and language, but also includes visual cognitive tools and the impact of learners on cognitive processes. In visual education, visual technology provides an abstract to a concrete processing method for educational activities and provides groundbreaking support for the forms and methods of education. When describing visual education, Fan Wengui also mentioned the role of technology in the process of visual education. He believes that visual education is the use of computer software and multimedia materials in the process of perception, cognition, imagination, and reasoning, by using simulation, simulation, and visualization to try to show it in the classroom. Ye Xindong also talked about visual guidance in his study. He believes that visual education is the study of the use of visual representations (such as graphic images and animated videos) and visual insights (such as mind maps, knowledge maps, and mind maps), and abstract educational content is materialized into the cognitive structure of learners.”.

AOverall, the current understanding of visual education pays more attention to the design and application of visual representation and visualization technology in the education process. This article understands visualization education from the perspective of developing visualization in scientific computing. Under the guidance of design methods, we use a series of visualization methods (such as concept maps and mind maps) to visualize abstract and complex knowledge, promote the understanding and application of knowledge, and finally help learners build cognitive structure teaching through activities.

Visual education combines the meaning of visual education with related scientific research and has the following characteristics: commitment to establishing a cognitive structure and knowledge system, strong initiative, effective visual behavior, accurate reflection, and evaluation. The main purpose of establishing cognitive structure is to grasp the meaning of target knowledge points. The meaning of a knowledge point is actually the connection between a knowledge point and another knowledge point. In traditional education, these connections are usually directly presented to students by teachers, but visualization education explores the visualization process through active and autonomous exploration and the use of visualization technology. On the one hand, visualization technology can alleviate the cognitive burden of students in creating meaningful work. On the other hand, through the personal experience of the process of meaning formation, people can gain a deeper understanding of knowledge, help the transfer and application of knowledge, and gradually improve their ability to form a cognitive structure.

Visual guidance can guide students to enter complex task situations for independent exploration, fully stimulate thinking, complete the preliminary construction of the knowledge network, and discover problem-solving strategies. Then teachers integrate sublimation (that is, optimize the knowledge network and improve problem-solving strategies). However, in traditional lecture-style classrooms, the content is usually presented by displaying and reviewing knowledge. Students only need to write down what the teacher has taught, and the students have very little room for thinking. Visual education requires students to understand, re-characterize, discuss, actively think and participate in classroom training.

The visualization process is especially important in visualization education. The process of visualization is the process of organizing ideas, and the result of visualization is the result of student learning. The learning outcomes here are not only related to the visualization of students' thinking, but also related to the improvement of students' thinking ability and intellectual growth. The core of visual education is that students repeatedly modify visual diagrams through active thinking and collaborative communication based on existing knowledge and experience to finally achieve learning results.

Reflection is the process of consciously exploring, analyzing, summarizing, evaluating, and coordinating cognitive activities. It is the basis for students to regulate learning and is the main form of enhancing self-awareness, self-monitoring, and self-regulation in the cognitive process. The most important thing in the visualization education process is the visualization process, and the most important thing in the visualization process is reflection. The reflection in the traditional classroom is usually the teacher summing up and reflecting on the content of the blackboard and presentation. In the visualization course, reflection is more flexible, and students can reflect on their own visualization process in a targeted manner.

## 4. University Political Video Course Development and Practice

### 4.1. University Political Video Course Model

**Figure 5:**
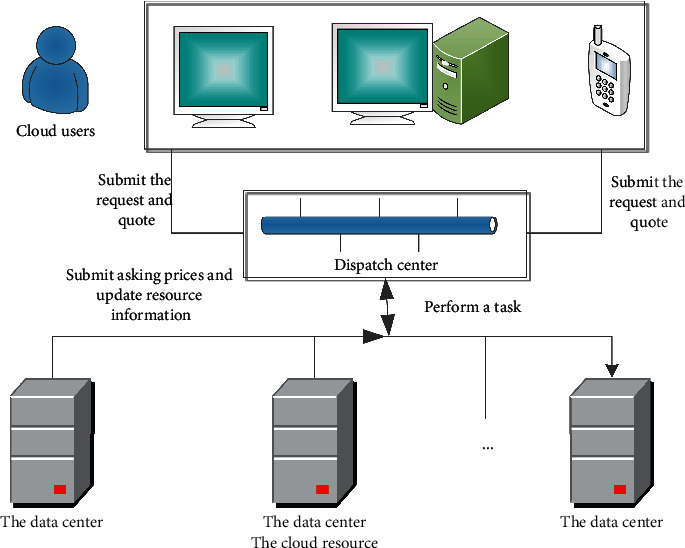
Cloud resource scheduling scheme model.

In the previous section, we have pointed out that university political video courses based on cloud services are essentially resource transactions between users and cloud service providers. There are two main ways of resource transactions in the existing cloud server market. One is that the cloud service provider allocates resources according to a fixed combination of resources and distributes them to users, but users' requirements for cloud resources are often flexible and changeable. Another cloud resource allocation method is based on the combined double auction resource allocation method. This method combines the needs and resource combinations of both parties in the transaction to make a quotation, which can realize multiple combinations of cloud resources and on-demand allocation of resources. This study proposes a combined double auction scheme of resources, which can comprehensively meet the diverse needs of users while protecting the interests of cloud service providers.  Figure 5 shows the model of the resource scheduling scheme proposed in this study. When the cloud service provider meets the user's needs, the user pays the cloud service provider, and the cloud service provider's revenue is mainly obtained from the fee paid by the user, while taking into account the cost of performing the user's task.

The cloud resource scheduling scheme proposed in this article mainly includes three parts.

*Users and Agents.* The agent creates a quote for the cloud service provider according to the resource requirements of the task being performed. At the same time, they must also consider the deadline of the task. If the cloud resources are not obtained before the deadline, the user will raise the quotation to obtain the cloud. Resources to complete the task.

Cloud service providers and their agents, the price of resources required by cloud service providers for their users mainly depends on storage and bandwidth. If a cloud service provider completes a task over time, it needs to pay a penalty for breach of contract. The penalty is proportional to the timeout period. The higher the user level, the more fines the cloud service provider needs to pay.

The auction agent sorts the offers according to the required resources and the offer price provided by the cloud service provider for the combined resources. If the user's quotation is met and the quotation is higher than the price provided by the cloud service provider, it will be combined based on the user's best quotation and the lowest quotation of cloud resources. Auction agents should also consider the priority of resources owned by different user levels.

### 4.2. Video Course Model Architecture

**Figure 6:**
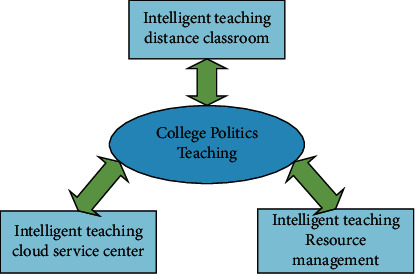
The overall architecture of university political video courses based on cloud services.

Considering the complex functions and high-performance requirements of the system, it is necessary to comprehensively consider the basic support platform, resources, system functions, and application platform functions to achieve a unified design, gradual refinement, and modularization to ensure the smooth implementation of the project and achieve the expected goals. Since the main technology adopted by this system is cloud service and SOA design mode, the overall requirement of the system is to integrate educational resources in the cloud and provide them in the form of services, and users of the education cloud can access the cloud anytime and anywhere according to their needs. Therefore, centering on the smart teaching cloud service system, the overall function of the system proposed in this study is shown in Figure 6.

It can be seen from the figure that the functions of the entire system are mainly divided into three parts: the smart teaching cloud service center, the smart teaching resource management, and the smart teaching remote classroom. The following is an introduction to each part in turn: The smart teaching cloud service center is mainly oriented to three roles: cloud service users, cloud service providers, and cloud platform managers. Cloud service users access the smart teaching cloud platform through terminals and use cloud services. The center inquires about the services it needs, the cloud service provider uploads the services that it can provide through the cloud service center, and the platform manager is responsible for reviewing the needs of users and managing the services provided by the cloud service provider. Smart teaching resource management is mainly oriented to two roles: cloud service users and cloud platform managers. For cloud service users, they can query, download, and use the teaching resources and electronic documents provided by the system through this smart teaching cloud platform. We can also upload your own resources, for example, pictures, documents, videos, audios, etc., to the platform to expand the resources of the platform; for cloud platform managers, it is necessary to conduct unified identity authentication for users and review and review resources. For authority management, it is needed to manage and integrate the security, effectiveness, and relevance of resources. Smart teaching remote classrooms are mainly for education cloud users, that is, students, teachers, and academic administrators. Teachers can upload teaching videos or teach online. Students can also listen to lessons online, complete homework and online exams, etc., provided all processes are available It is completed through the Internet, and the educational administration staff can arrange the syllabus, specify the curriculum plan, enter and analyze the test scores according to the actual situation.

### 4.3. Functional Design

According to the overall structural design of the above system and analysis from the perspective of system functions, the main user roles of the university political video courses based on cloud services proposed in this article are divided into two categories, one is the administrator of the smart teaching cloud platform, and the other is smart teaching. The users of cloud platform mainly include students, teachers, and education administrators. The Smart Teaching Cloud Service Center is the core of the entire university's political video courses and an important interaction center between service providers and service consumers. In the university political video course cloud platform designed in this article, if education cloud users want to obtain the services they need, they must complete user registration and login in order to find the services or resources they need, and rent them on demand through payment. In terms of corresponding services and resources, for service providers, they need to register the services they can provide through the cloud service center, and upload relevant text, picture introductions, and other description documents; for cloud platform administrators, users of all platforms conduct user management and complete the tasks of service management and service monitoring.

Smart teaching resource management is equivalent to a huge data warehouse stored in the cloud. Here, cloud service users can realize the storage, download, upload, and other functions of educational resources, while the review, classification, and integration of many resources require the cloud platform. The manager will do it. The function of smart teaching in remote classrooms is the best embodiment of the realization of the entire system. The user roles are divided into students, teachers, and academic administrators. For teachers and students, teachers can choose whether to record the course as a video, or upload it to the cloud service platform for smart teaching or online live broadcast. At the same time, the service can also provide online Q&A and discussion, online exams and other functions, and students can complete for exams, teachers can complete exam management functions such as reviewing test papers and giving scores.

For academic administrators, they can manage the personal information of teachers and students, arrange for teachers to manage their own courses, students choose courses according to their needs, and at the same time formulate teaching plans and syllabuses, students complete a course and complete the course exam. After that, the teacher enters the test results, and after the administrator completes the review, the students can inquire about their own scores and rankings through the system terminal.

### 4.4. Database Design

University political video courses based on cloud services are mainly a teaching system for cloud courses. The main users are cloud service users, cloud service providers, and cloud platform managers. At the same time, cloud service users need to learn, and cloud service providers need to provide corresponding services, and administrators need to manage these users. In order to meet these needs, the university political video courses based on cloud services include user tables, role tables, curriculum tables, permission tables, homework tables, and user homework tables. The data storage of the system described in this article uses MySQL data tables for storage. There are a total of five tables for the university political video courses based on cloud services, including user table, role table, curriculum table, permission table, assignment table, and user assignment table. The following describes the tables in thedatabase indetail:

1. User table (user_info): the main function of the user table is to record user information, mainly including user number, user name and password, user authority number, and user role number. The specific information is shown in Table 1:
2. Role table (role_info): the role table is mainly used to record user role information, including role numbers. The specific role table information is shown in Table 2:
3. Course schedule (course_info): the main function of the course schedule is to record course information, including course number, course information, and start time. The specific curriculum information is shown in Table 3:
4. Permission table (permission_info): the main function of the permission table is the information on user permissions, mainly including permission number and permission name. The specific permission table information is shown in Table 4:

## 5. Conclusion

In this study, the wireless sensor network is used as the research background to study the important aspects of multichannel MAC protocol, multichannel spectrum acquisition, multichannel broadcasting, and multichannel communication related to wireless sensor network time synchronization protocol security for the research of multichannel communication technology. There are still many shortcomings. In this study, a new multichannel MAC-RTMAC protocol based on the parallel negotiation mechanism protocol is developed. In this protocol, the node uses a pseudo-random sequence to generate the node's standard channel switching sequence, and the node switches between multiple channels according to this sequence. At the same time, the duration of the node is divided into several time slices. The node pseudo-randomly selects a time slice, receives information in the selected time slice, and sleeps in the remaining time slices. The protocol uses a distributed planning algorithm. The sending node jumps to the standard channel of the receiving node, and communicates according to the standard channel switching sequence of the receiving node. In order to find a better correspondence between the classroom environment and visual education in the future, this article first introduces the research status of visual education, summarizes the characteristics of visual education, and the characteristics of some typical and commonly used visualization technologies. Next, from the perspective of the future classroom, the current research status of the future classroom is introduced, and the visual environment structure of the future classroom is elaborated. On this basis, combined with related theories, the characteristics of visual teaching are summarized in the future classroom environment.

## Figures and Tables

**Table 1:** User table.

Field name	Description	Type of data	Is it empty	Key
Userid	User ID	Iiit	N	Primary key
Username	Account name	varchar (20)	N	—
Password	Password	varchar (20)	N	—
Permissionid	Authority number	Iiit	N	—
Roleid	Role number	Iiit	N	—

**Table 2:** Role table.

Field name	Description	Type of data	Is it empty	Key
Roleid	Numbering	Iiit	N	Primary key
Rolename	Role name	Varchar (20)	N	—

**Table 3:** Course schedule.

Field name	Description	Type of data	Is it empty	Key
Userid	Numbering	Iiit	N	Primary key
Coursename	Course title	Varchar (20)	N	—
Date	Start time	Date	N	—

**Table 4:** Permission table.

Field name	Description	Type of data	Is it empty	Key
Roleid	Numbering	Iiit	N	Primary key
Rolename	Role name	Varchar (20)	N	—

## Data Availability

The data used to support the ﬁndings of this study are available from the corresponding author upon request.

## References

[1] Jianzhong Z., Radio G. (2018). Design and implementation of smartphone broadcast system based on wireless. *Radio & TV Broadcast Engineering*.

[2] Qin Y. (2019). Transmission reliability of wireless communication system-based on optical fiber signal processing. *Journal of Optical Communications*.

[3] Li A., Spano D., Krivochiza J. (2020). A tutorial on interference exploitation via symbol-level precoding: overview, state-of-the-art and future directions. *IEEE Communications Surveys & Tutorials*.

[4] Dai L., Wang Z., Yang Z. (2012). Next-generation digital television terrestrial broadcasting systems: key technologies and research trends. *IEEE Communications Magazine*.

[5] Tanougast C., Rihani M.-Al-F., Seyedi M. H. (2019). “Recent advances in wireless communication. *Recent Advances in Wireless Communication*.

[6] Raheb K. E., Stergiou M., Katifori A., Ioannidis Y. (2020). Dance interactive learning Systems. *ACM Computing Surveys*.

[7] Narita F. M. (2017). Informal learning in action: the domains of music teaching and their pedagogic modes. *Music Education Research*.

[8] Song R. (2021). Research on the application of computer multimedia music system in college music teaching. *Journal of Physics: Conference Series*.

[9] Ivanov Y., Bobick A. (2000). Recognition of visual activities and interactions by stochastic parsing. *IEEE Transactions on Pattern Analysis and Machine Intelligence*.

[10] Xu X., Li D., Sun M. (2019). Research on key technologies of smart campus teaching platform based on 5G network. *IEEE Access*.

[11] Jianzhong Z. (2017). Design and implementation of smartphone broadcast system based on wireless network technology. *Radio & TV Broadcast Engineering*.

[12] Barzegar H. R., Reggiani L., Dossi L. Extending the range of full-duplex radio with multi-carrier partial overlapping.

[13] Gpp (2009). Evolved universal terrestrial radio access (E-UTRA); multiplexing and channel coding. *3rd Generation Partnership Project (3GPP), Technical Specification (TS)*.

[14] Gamage H., Rajatheva N., Latva-aho M. Channel coding for enhanced mobile broadband communication in 5G systems.

[15] Wu X., Zhao C., You X., Li S. (2007). Robust diversity-combing receivers for LDPC coded FFH-SS with partial-band interference. *IEEE Communications Letters*.

[16] AlAmmouri A., ElSawy H., Amin O., Alouini M.-S. (2016). In-band *α*-duplex scheme for cellular networks: a stochastic geometry approach. *IEEE Transactions on Wireless Communications*.

